# A temporal cluster of acute promyelocytic leukemia

**DOI:** 10.17179/excli2022-5676

**Published:** 2023-01-05

**Authors:** Stephen E. Langabeer

**Affiliations:** 1 Cancer Molecular Diagnostics, St. James's Hospital, Dublin, Ireland

## ⁯⁯

Acute promyelocytic leukemia (APL) is a subtype of acute myeloid leukemia (AML) that possesses a typical morphology, and in greater than 98 % of cases, the t(15;17) translocation that results in the *PML-RARA* fusion oncogene. Current therapeutic options include regimens that include all-trans retinoic acid and arsenic trioxide that directly target the underlying molecular abnormalities of APL (Sanz and Barragán, 2021[9]). The incidence of APL in adults has been previously estimated at between two and six cases per 10 million people per year (Mele et al., 1995[8]; Chen et al., 2012[3]). Early in 2022, an increase in newly presenting cases of APL was noted at a central laboratory for leukemia molecular diagnostics that possibly represented a temporal cluster. In order to ascertain the nature of this increase in new patient numbers and additionally to estimate the incidence of this type of leukemia in the Republic of Ireland, an historical audit was performed for molecularly characterized APL. 

**Figure 1:**
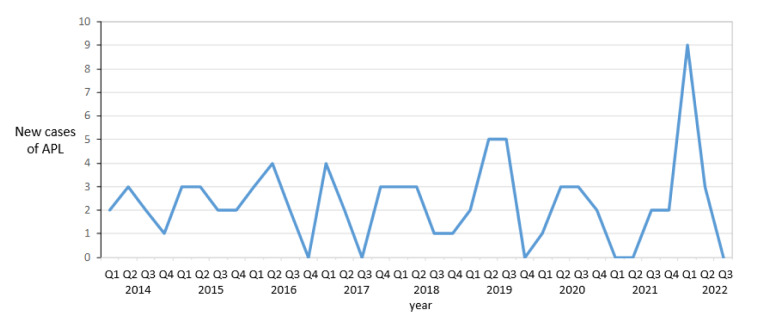
Quarterly incidence of newly diagnosed acute promyelocytic leukemia (APL) from Q1 2014 to Q3 2022

A database search was performed for all new cases of *PML-RARA*-positive APL at a central laboratory for leukemia molecular diagnostics from January 2014 to end quarter three (Q3) September 2022 inclusive. *PML-RARA* transcripts were detected by a standardized real-time quantitative polymerase chain reaction approach (Gabert et al., 2003[5]). Eighty-one cases of newly diagnosed *PML-RARA*-positive APL were identified within the audit period of whom 40 were male and 41 were female (median age 51 years; range 1-91 years). The distribution of *PML-RARA* breakpoint cluster (bcr) types was bcr1 n=34, bcr2 n=8 and bcr3 n=39. Cases were grouped into three-monthly quarters with a mean of 8.5 cases per year (equivalent to 2.125 cases per quarter) from Q1 2014 to Q4 2021. The Poisson distribution probability, assuming 2.125 cases per quarter, would suggest that quarters in which there are six (p = 0.006), seven (p = 0.002), or more cases are statistically improbable to happen by chance alone: a conspicuous peak of nine new cases was observed in Q1 of 2022 (Figure 1(Fig. 1)). These nine cases comprised five males and four females with a median presentation age of 52 years (range 24-74 years) with bcr1 (n=2), bcr2 (n=1) and bcr3 (n=6).

During the eight years prior to 2022, the mean incidence of molecularly detected APL in the Republic of Ireland was eight or nine cases per year, higher than that previously reported in alternative, historical, adult cohorts and geographical locations. Reasons may include the inclusion of pediatric APL cases in this study and the heightened awareness of relevant testing given high response rates with modern therapy. Estimating the annual incidence of APL has not only diagnostic implications but will aid in planning treatment services.

An explanation for the temporal clustering within Q1 2022 is not immediately apparent: the demographics of the nine patients were representative of the whole cohort and were from disparate locations. It is acknowledged that this brief report has shortcomings associated with reporting cancer clusters (Coory and Jordan, 2013[4]). Geographical clustering of APL has been previously reported but little or no association demonstrated with respect to race, gender, additional cytogenetic abnormalities, additional mutations or upper respiratory viral illness (Brunner et al., 2018[1]; Li et al., 2020[7]). Evidence for seasonal variation in both APL and AML in general has also been documented (Calip et al., 2013[2]; Hassan et al., 2021[6]), prompting speculation of an environmental factor: identification of which might enlighten understanding of APL etiology and strategies for possible prevention.

## Declaration

### Conflict of interest

The authors declare that they have no conflicts of interest.

### Acknowledgments

The author acknowledges the contribution of those members of the Cancer Molecular Diagnostics department involved in molecular testing over the audit period.
